# Transient ST-segment elevation myocardial infarction without flow-limiting obstructions in the epicardial coronary arteries on angiography: coronary artery vasospasm, myocardial infarction with non-obstructive coronary arteries or spontaneous coronary artery dissection?

**DOI:** 10.1093/ehjimp/qyae104

**Published:** 2024-10-12

**Authors:** Sahrai Saeed, Trond Cooper, Anne Margrethe Tveit, Svein Rotevatn, Terje H Larsen

**Affiliations:** Department of Heart Disease, Haukeland University Hospital, 5021 Bergen, Norway; Department of Cardiology, Stavanger University Hospital, Stavanger, Norway; Department of Heart Disease, Haukeland University Hospital, 5021 Bergen, Norway; Department of Heart Disease, Haukeland University Hospital, 5021 Bergen, Norway; Department of Heart Disease, Haukeland University Hospital, 5021 Bergen, Norway; Department of Biomedicine, University of Bergen, Bergen, Norway

**Keywords:** Acute coronary syndrome, Multimodality imaging, Cardiac MRI, Echocardiography, Interventional cardiology

A 42-year-old previously healthy man was admitted to our hospital following an episode of acute chest pain while driving. ECG performed out-of-hospital showed ST-segment elevation in leads V5–V6 and reciprocal changes in leads V1–V4 (*Figure 1A*). Nitroglycerine, aspirin, clopidogrel, and 5 mg morphine were administered prior to admission. On arrival, his chest pain spontaneously resolved and the ST-segment changes reverted to nearly normal (*Figure 1B*). Coronary angiography did not show any flow-limiting stenosis in the right coronary artery (*Figure 1C*) and left anterior descending artery (*Figure 1D*), but a mild plaque in the proximal left circumflex (LCx) artery (*Figure 1E*, arrow). Troponin T increased from 18 ng/L (ref. < 15 ng/L) at presentation to 572 ng/L 4 h later. Echocardiography showed normal systolic left ventricular function (ejection fraction 60% and global longitudinal strain −19%) (*Figure 1*F), with no signs of regional wall motion abnormalities. The basal segments in the posterolateral wall on bull’s eye plot had impaired strain values (*Figure 1F*, arrows), with significant post-systolic shortening (data not shown). A transient occlusion of the obtuse marginal branch of the LCx was suspected. This was verified by a cardiac magnetic resonance (CMR) the following day, demonstrating signs of oedema on STIR (short T1 inversion recovery) sequence (*Figure 1G*, arrows, short-axis) and ischaemic injury as reflected by late gadolinium enhancement (*Figure 1H*, arrows, short-axis), with clear delineation from adjacent healthy myocardium in the mid-lateral wall (shown in T2 mapping sequence short-axis with arrows in (*Figure 1I*). A CT coronary angiogram excluded spontaneous coronary artery dissection (SCAD)/subintimal haematoma, although SCAD is typically a less common cause of acute coronary syndrome in men. The CT did however confirm a soft (low attenuation) plaque in the proximal LCx (*Figure 1J*, arrow). The patient was discharged on Day 3 with antiplatelet therapy and a statin. Our case illustrates the usefulness of multimodality imaging performed in a logical manner, prompting immediate and accurate diagnosis and correct pathway of care. We illustrated an aborted infarct leading to transient occlusion of the obtuse marginal branch of the LCx artery, which was suggestive of MINOCA (myocardial infarction with non-obstructive coronary arteries). Utilizing CMR, as well as other non-invasive imaging, is currently recommended by the 2023 European Society of Cardiology guidelines for the management of acute coronary syndromes, especially if the underlying cause of acute myocardial injury/MINOCA is not established by conventional coronary angiography. The main emphasis in the diagnostic work-up is differentiating MINOCA presenting as type 1 myocardial infarction from type 2 myocardial infarction. This has important clinical implications to provide optimal and appropriate therapy (i.e. calcium channel blockers for coronary spasm and antiplatelet and antiatherosclerosis drugs in type 1 injury).

**Figure 1.**
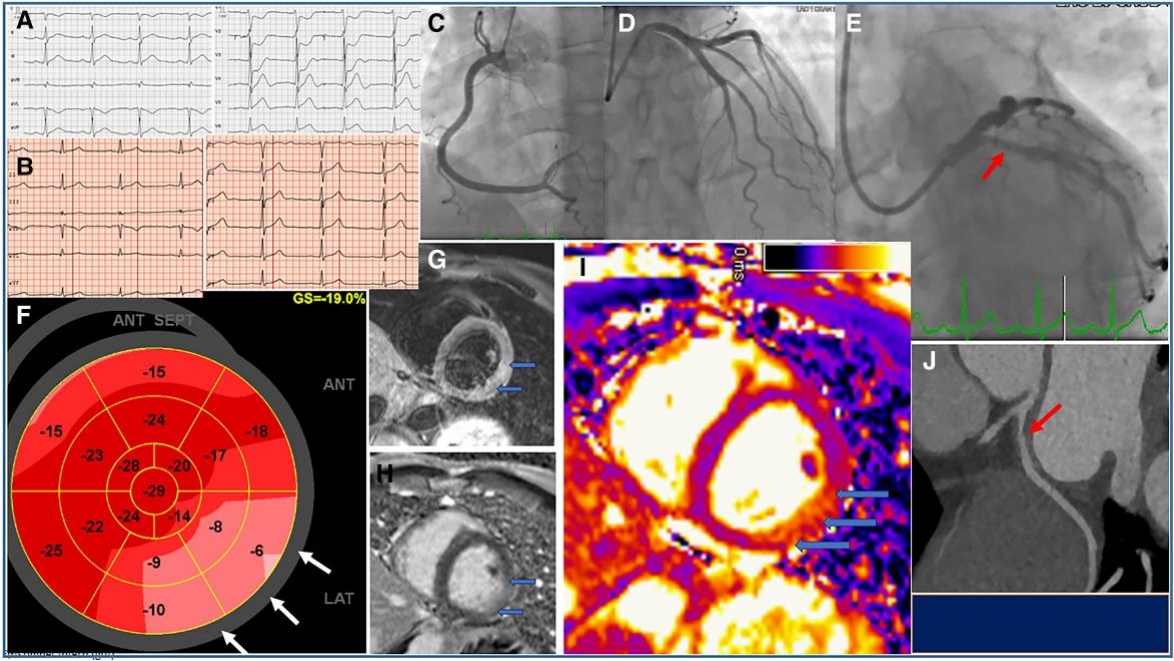
(*A*) ECG performed out-of-hospital showed ST-segment elevation in leads V5–V6 and reciprocal changes in leads V1–V4. (*B*) ECG recorded at arrival showed regression of the ST-segment changes to nearly normal. (*C* and *D*) Coronary angiography showing open right coronary and left anterior descending artery. (*E*) Coronary angiography showing mild plaque in the proximal left circumflex artery. (*F*) Echocardiography bull's eye plot showing reduced strain values in the basal posterolateral segment. (*G*) Cardiac MRI STIR showing oedema and presumed ischaemic injury on LGE. (*I*) T2 mapping on MRI showing injury. (*J*) CT coronary angiogram showing a soft plaque in the left circumflex artery.

A limitation is that coronary angiography was performed prior to the subsequent troponin rise and intracoronary imaging (intravascular ultrasound and intracoronary optical coherence tomography) was unfortunately not performed. Similarly, provocative test to exclude coronary artery spasm was not performed, and the final diagnosis of transient occlusion of the marginal branch is based upon CMR findings. However, CMR can identify the underlying cause of acute myocardial injury in the vast majority of patients (up to 87%) presenting with MINOCA, and should be performed as soon as possible during the index hospitalization in order to enhance the diagnostic yield.

**Consent:** Consent was obtained from the patient for presenting this image focus.

**Funding:** No funding was received for this case study.

**Data availability:** The data underlying this article will be shared on reasonable request to the corresponding author.

## Lead author biography


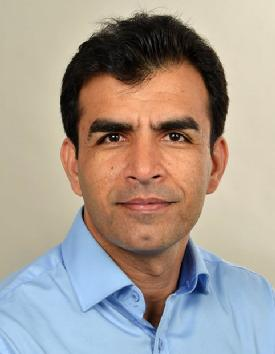


Dr Sahrai Saeed, MD, PhD, FESC is a senior consultant cardiologist and clinical researcher at Haukeland University Hospital in Bergen, Norway. His special interest includes echocardiography and multimodality imaging.

